# Comparative metabolomic analysis reveals the variations in taxoids and flavonoids among three *Taxus* species

**DOI:** 10.1186/s12870-019-2146-7

**Published:** 2019-11-29

**Authors:** Ting Zhou, Xiujun Luo, Chengchao Zhang, Xinyun Xu, Chunna Yu, Zhifang Jiang, Lei Zhang, Huwei Yuan, Bingsong Zheng, Erxu Pi, Chenjia Shen

**Affiliations:** 1 College of Life and Environmental Sciences, Hangzhou Normal University, Hangzhou, 310036 China; 2 Key Laboratory for Quality and Safety of Agricultural Products of Hangzhou City, College of Life and Environmental Science, Hangzhou Normal University, Hangzhou, 310036 China; 3 Zhejiang Provincial Key Laboratory for Genetic Improvement and Quality Control of Medicinal Plants, Hangzhou Normal University, Hangzhou, 310036 China; 4 Department of Plant Pathology, Washington State University, Pullman, WA 99164-6430 USA; 5 State Key Laboratory of Subtropical Silviculture, Zhejiang A & F University, Hangzhou, 311300 People’s Republic of China; 6 Center for Cultivation of Subtropical Forest Resources (CCSFR), Zhejiang A & F University, Hangzhou, 311300 People’s Republic of China

**Keywords:** Metabolome, Interspecific differential accumulation, Systematic correlativity analysis, Taxoid, *Taxus*

## Abstract

**Background:**

Trees of the genus *Taxus* are highly valuable medicinal plants with multiple pharmacological effects on various cancer treatments. Paclitaxel from *Taxus* trees is an efficient and widely used anticancer drug, however, the accumulation of taxoids and other active ingredients can vary greatly among *Taxus* species. In our study, the metabolomes of three *Taxus* species have been investigated.

**Results:**

A total of 2246 metabolites assigned to various primary and secondary metabolic pathways were identified using an untargeted approach. Analysis of differentially accumulated metabolites identified 358 *T. media*-, 220 *T. cuspidata*-, and 169 *T. mairei*-specific accumulated metabolites, respectively. By searching the metabolite pool, 7 MEP pathway precursors, 11 intermediates, side chain products and derivatives of paclitaxel, and paclitaxel itself were detected. Most precursors, initiated intermediates were highly accumulated in *T. mairei*, and most intermediate products approaching the end point of taxol biosynthesis pathway were primarily accumulated in *T. cuspidata* and *T. media*. Our data suggested that there were higher-efficiency pathways to paclitaxel in *T. cuspidata* and *T. media* compared with in *T. mairei*. As an important class of active ingredients in *Taxus* trees, a majority of flavonoids were predominantly accumulated in *T. mairei* rather than *T. media* and *T. cuspidata*. The variations in several selected taxoids and flavonoids were confirmed using a targeted approach.

**Conclusions:**

Systematic correlativity analysis identifies a number of metabolites associated with paclitaxel biosynthesis, suggesting a potential negative correlation between flavonoid metabolism and taxoid accumulation. Investigation of the variations in taxoids and other active ingredients will provide us with a deeper understanding of the interspecific differential accumulation of taxoids and an opportunity to accelerate the highest-yielding species breeding and resource utilization.

## Background

Taxol (generic name paclitaxel) is the major bioactive component of the *Taxus* species widely used in the treatment of various cancers, such as ovarian cancer, breast cancer and squamous cancers [1]. Since its approval for ovarian cancer treatment in 1992, the demand for paclitaxel and its derivatives has increased [2]. Several barriers, including low content of taxoids, exhausted natural resources and high loss rate of purification, limited the increasing of paclitaxel supply. Therefore, extraction of its analogues and/or derivative is an alternative economic solution for the production of paclitaxel at an industrial level [3].

Production of paclitaxel and other taxoids is improved by increasing knowledge of the paclitaxel biosynthetic pathway, thus the pathway becoming the principal object of many studies [4, 5]. The whole paclitaxel biosynthetic pathway produces a large number of precursors, intermediates and derivatives of paclitaxel [6, 7]. Firstly, the precursor of the diterpenoid taxane core geranylgeranyl diphosphate (GGPP) is synthesized using three units of the C_5_ isoprenoid precursors isopentenyl diphosphate (IPP) and one unit of dimethylallyl diphosphate (DMAPP), which are supplied by the plastidial 2-C-methyl-D-erythritol phosphate (MEP) pathway [8, 9]. The key enzyme taxadiene synthase (TS) catalyzes GGPP to yield the taxane skeleton taxa-4(5),11(12)-diene [10, 11]. Then, a series of hydroxylation, acetylation and *N*-benzoylation of taxane skeleton produce a number of intermediates in the pathway towards paclitaxel. For example, acetylation of 10-deacetylbaccatin-III (10-DAB) produces baccatin III, an advanced intermediate for paclitaxel biosynthesis [2, 12]. The assembly of C13-side chain appended to baccatin III (BAC) to form *N*-debenzoyl-2′-deoxytaxol is considered as the final step of the paclitaxel biosynthesis pathway [13].

In addition to paclitaxel, more than 500 taxoid secondary metabolites are contained in different species of the genus *Taxus* [14–16]. For example, paclitaxel and three related taxoids, 10-DAB III, BAC, and cephalomannine, were extracted from the needles of *Taxus cuspidata*, *Taxus chinensis*, and *Taxus media* [17]. In *Taxus mairei*, most known taxoids were also determined by high-performance liquid chromatography-tandem mass spectrometry (HPLC-MS/MS) [18]. Additionally, three new taxoids, along with three known taxoids, were isolated from the seeds of *T. cuspidata* [19]. All *Taxus* species produce paclitaxel; however, the level of accumulated taxoids can vary significantly [20]. A large-scale analysis of the taxoid concentrations revealed that no single species contained the highest levels of all the metabolites [21].

An untargeted metabolome provides a good opportunity to systematically analyze primary and secondary metabolites, as well as to identify potential unknown compounds in plants [22]. In the *Taxus* genus, the first metabolomic analysis was published in 2003, profiling the metabolites of *T. media* cultures induced by a MeJA treatment [23]. A metabolomic approach with LC-IT-TOF-MS was used to investigate the variations in taxoid biosynthesis in cultured seedlings of *T. mairei* [24]. Recently, an integrated proteomic/metabolomic approach revealed that a short-term high dose of ultraviolet-A radiation could increase paclitaxel production in *T. mairei* [25].

Recent technical advances in the large-scale identification of metabolites have revealed the complex processes involved in regulating plant metabolism [26, 27]. So far, in the *Taxus* genus, 14 species and cultivars with varied levels of taxoids have been identified [28, 29]. Investigation of the variations in taxoids and other metabolites will provide us an opportunity to accelerate the highest-yielding species breeding.

## Results

### Untargeted metabolite profiling the metabolomes of different Taxus species

**Fig. 1:**
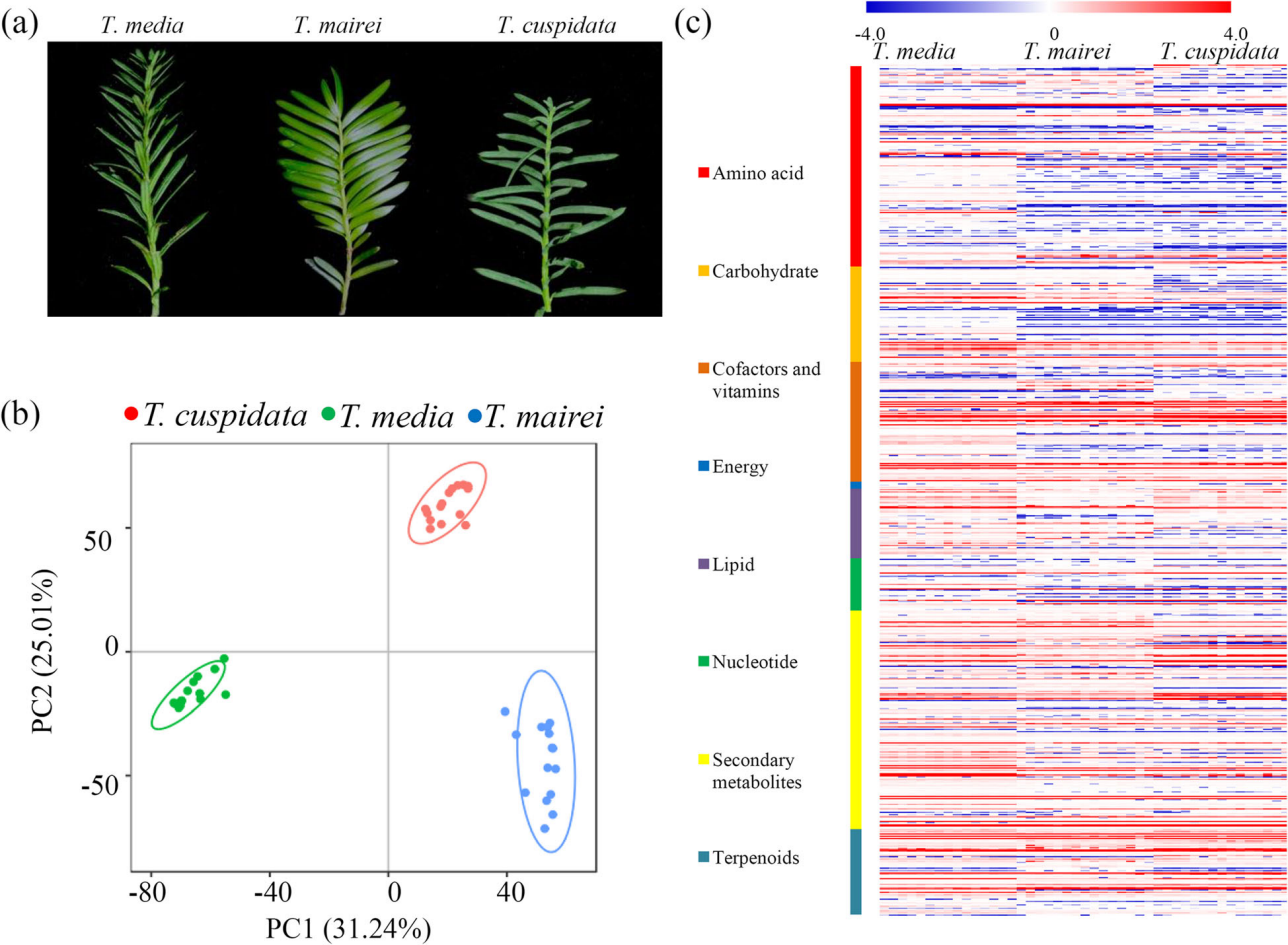
Untargeted metabolite profiling identifies the metabolites in the tested *Taxus* trees. **a** A picture of *T. media*, *T. mairei* and *T. cuspidata* under greenhouse condition. Fresh twigs were harvested from three cultivated *Taxus* species. **b** The PCA data of the samples from three different species. The red spots indicated the samples from *T. cuspidata*; the green spots indicated the samples from *T. media;* and the blue spots indicated the samples from *T. mairei*. **c** A heatmap of the metabolites grouped by Kyoto Encyclopedia of Genes and Genomes pathway found in the metabolomes of the three *Taxus* species (*n* = 15). The heatmap scale ranges from − 4 to + 4 on a log_2_ scale

To explore the comprehensive variations in metabolomes of different *Taxus* species, an untargeted approach (15 repeats for each group) was applied, identifying 2246 metabolites from 8712 ions with a relative standard deviation < 30% (Additional file 1). Similar to the differences in twig morphology, variations in the metabolomes among different *Taxus* species were also observed (Fig. 1a). For quality checking, total ion chromatograms were generated, suggesting that the sample preparation met the common standards (Additional file 2). To produce an overview of the metabolic variations, a PCA was performed, and the percentages of explained value in the metabolome analysis of PC1 and PC2 were 25.01 and 31.24%, respectively. The PCA data showed three clearly separated sample groups, indicating separations among the three different species (Fig. 1b). Based on their KEGG annotations, 747 metabolites were predicted to be involved in various primary metabolic pathways, including the amino acid-, carbohydrate-, cofactor and vitamin-, energy-, lipid-, nucleotide-, secondary metabolite-, and terpenoid-related pathways (Fig. 1c and Additional file 3).

### Clustering of differential accumulated metabolites

**Fig. 2:**
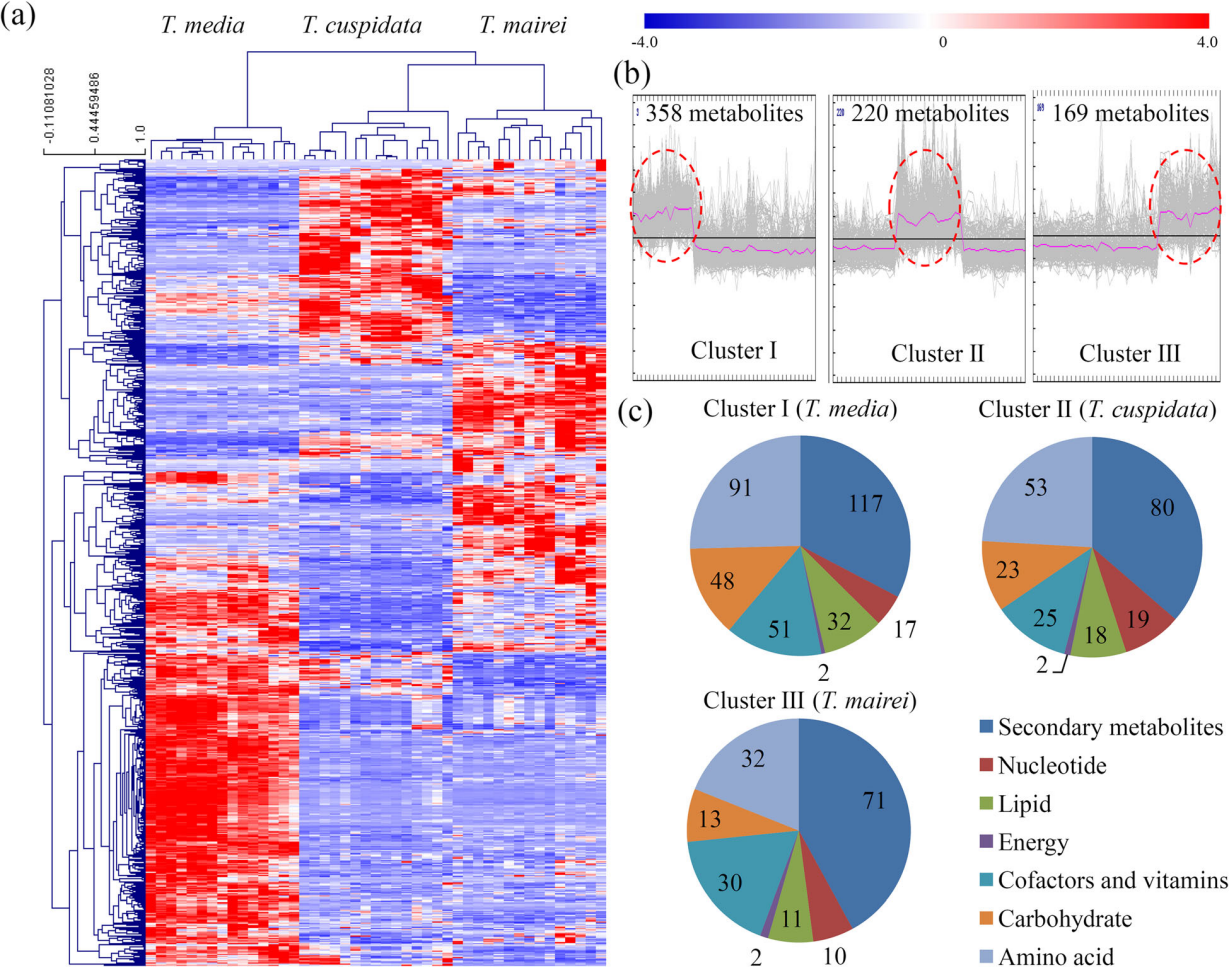
The variations in the metabolites among three *Taxus* species. **a** A heatmap of the relative amounts of DAMs from the three different species. **b** Clustering of the DAMs into three Clusters. Red cycles indicated the species specific accumulated metabolites. **c** These DAMs were also assigned into various primary metabolic categories

All annotated metabolites were clustered to identify the differential accumulated metabolites (DAMs) among three *Taxus* species (Fig. 2a). All DAMs were grouped into three Clusters: I, II and III. The *T. media* predominantly accumulated metabolites were grouped into Cluster I (358 metabolites), the *T. cuspidata* predominantly accumulated metabolites were grouped into Cluster II (220 metabolites), and the *T. mairei* predominantly accumulated metabolites were grouped into Cluster III (169 metabolites) (Fig. 2b). Our data showed that the DAMs belonging to the ‘secondary metabolites’, ‘lipids’, ‘cofactors and vitamins’, ‘carbohydrate’ and ‘amino acid’ categories were predominantly accumulated in *T. media* (Fig. 2c). The Cluster I (*T. media* predominantly accumulated) consisted of 117 secondary metabolites, 91 amino acids, 51 cofactors and vitamins, 48 carbohydrates, 32 lipids, 17 nucleotides and 2 energy-related metabolites; the Cluster II consisted of 80 secondary metabolites, 53 amino acids, 25 cofactors and vitamins, 23 carbohydrates, 18 lipids, 19 nucleotides and 2 energy-related metabolites; and the Cluster III consisted of 71 secondary metabolites, 32 amino acids, 30 cofactors and vitamins, 13 carbohydrates, 11 lipids, 10 nucleotides and 2 energy-related metabolites (Fig. 2c).

To get a comprehensive overview of variations, all DAMs were classified into different known metabolic pathways. In total, 32, 29, and 38 major pathways were enriched in the *T. mairei* vs *T. cuspidata* (Additional file 4), *T. media* vs *T. mairei* (Additional file 5), and *T. media* vs *T. cuspidata* (Additional file 6) comparisons. Interestingly, the largest number of DAMs in each comparison were enriched in the ‘diterpenoid biosynthesis’ pathway.

### Variations in the abundance levels of taxoids among three *Taxus* species

**Fig. 3:**
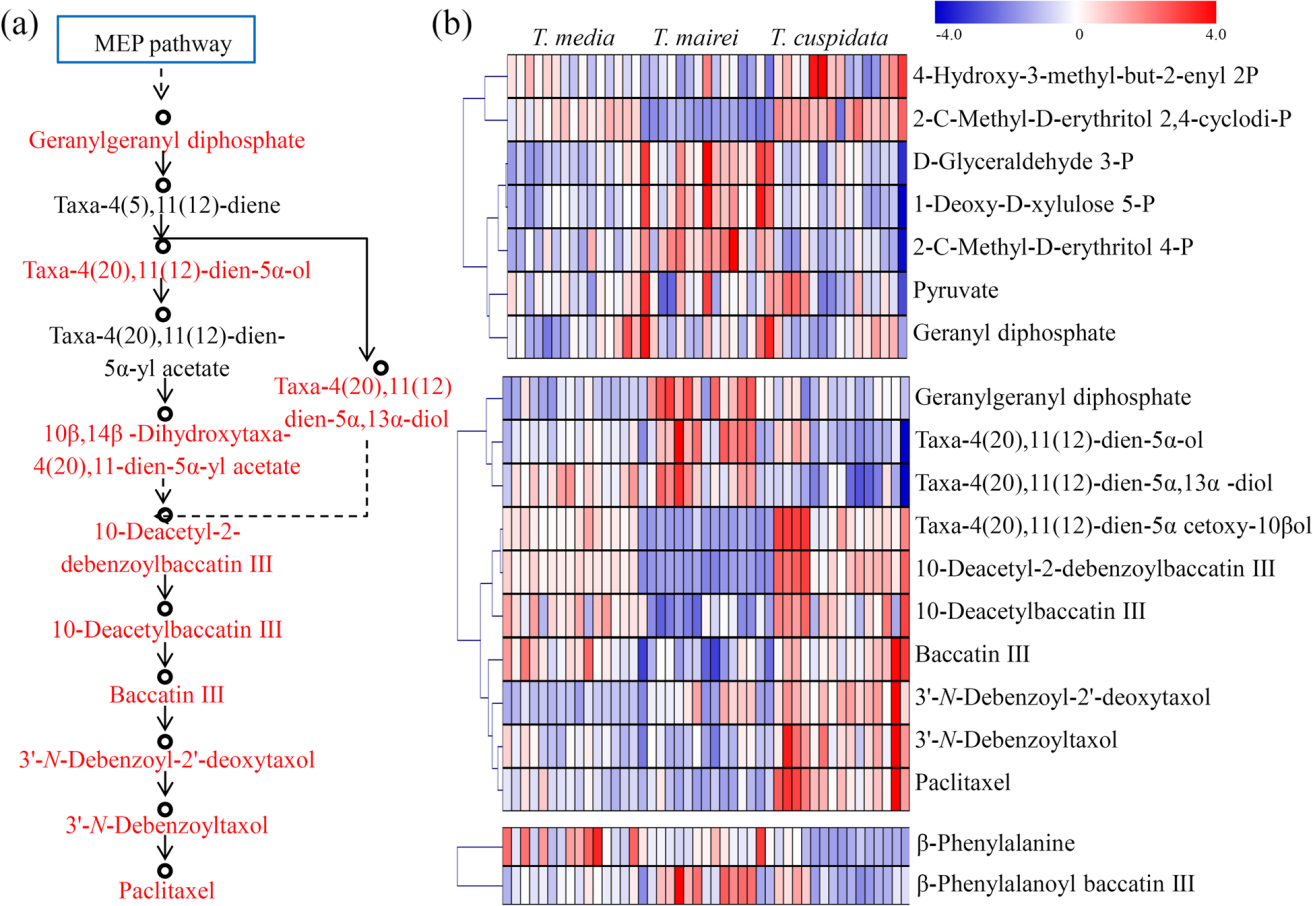
Analysis of the relative amounts of taxoids in the Taxus metabolomes from the three different species. (**a**) Overview of the taxol biosynthesis pathway. (**b**) The relative accumulation of taxoids, intermediates and derivatives in the three different species. The heatmap scale ranges from -4 to +4 on a log_2_ scale

**Fig. 4:**
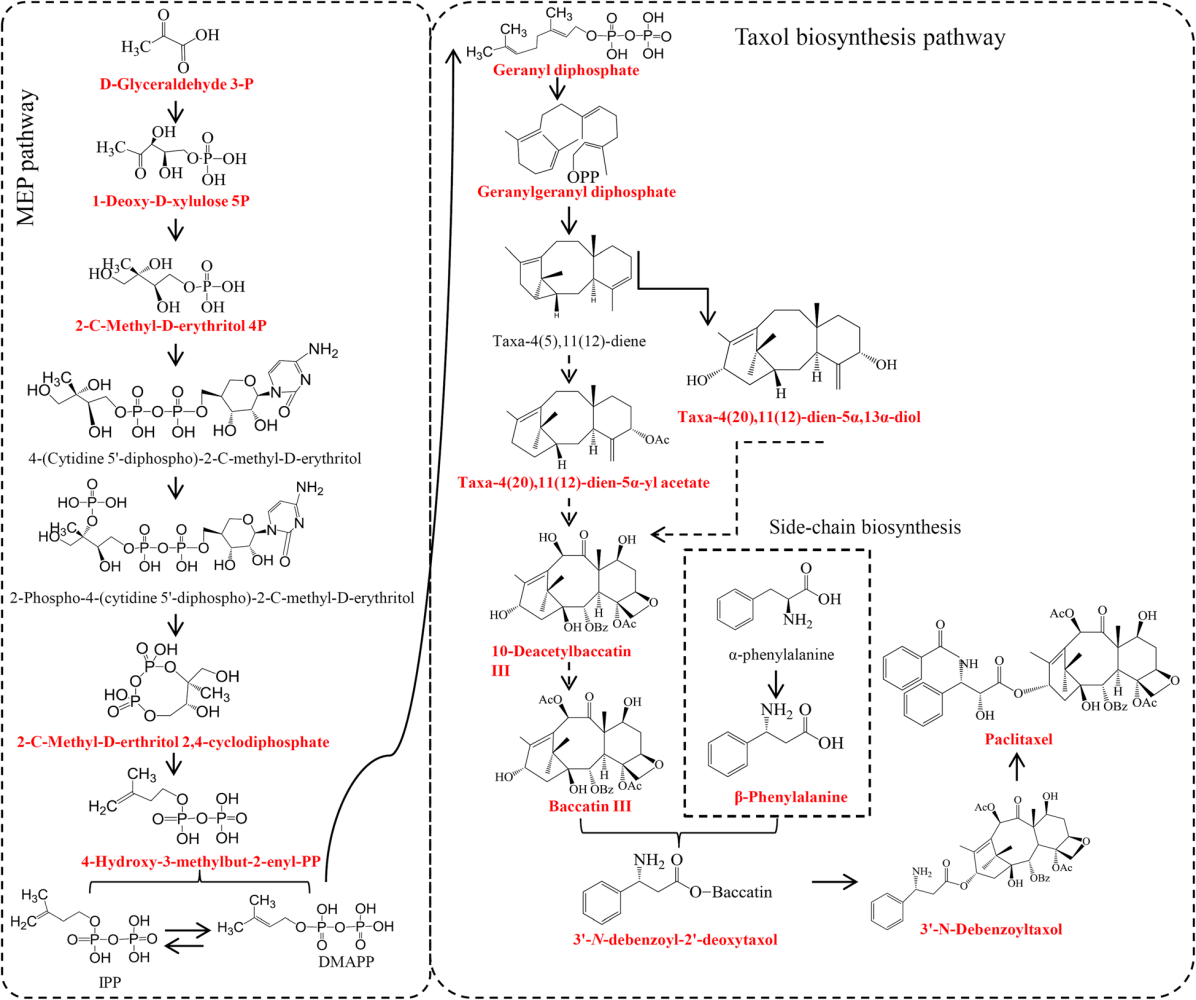
The complete biosynthetic pathway of taxol. The red font indicated the taxane precursors whose structure has been determined in the present study

Paclitaxel biosynthesis is an intricate metabolic pathway that involves a number of precursors, intermediates, and derivatives [5, 30]. By searching the metabolite pool, seven precursors from the MEP pathway, nine intermediates and derivatives, two side chain products, and paclitaxel were detected (Fig. 3a). For the MEP pathway, several precursors, such as D-glyceraldehyde 3-phosphate, 1-deoxy-D-xylulose 5-phosphate, and 2-C-methyl-D-erythritol 4-phosphate, were predominantly accumulated in *T. mairei*. Two precursors, 4-hydroxy-3-methyl-but-2-enyl diphosphate and 2-C-methyl-D-erythritol 2,4-cyclodiphosphate, were significantly accumulated in *T. cuspidata*. For the intermediate and derivative products, GGPP, Taxa-4(20),11(12)-dien-5α-ol, and Taxa-4(20),11(12)-dien-5α,13α-diol were predominantly accumulated in *T. mairei*; Taxa-4(20),11(12)-dien-5α cetoxy-10β ol, 10-Deacetyl-2-debenzoylbaccatin III, 10-Deacetylbaccatin III, and Baccatin III were highest in *T. mairei* and *T. media*; and 3′-*N*-Debenzoyl-2′-deoxytaxol, 3′-*N*-Debenzoyltaxol, and Paclitaxel were predominantly accumulated in *T. cuspidata*. For the side chain products, β-Phenylalanine was highly accumulated in *T. media* and β-Phenylalanoyl baccatin III was greatly accumulated in *T. mairei* (Fig. 3b). The complete biosynthetic pathway, including the elucidated and putative steps, was summarized in Fig. 4. All the taxane precursors that has been determined in our study were highlighted.

### Variations in the abundance levels of flavonoids among three *Taxus* species

**Fig. 5:**
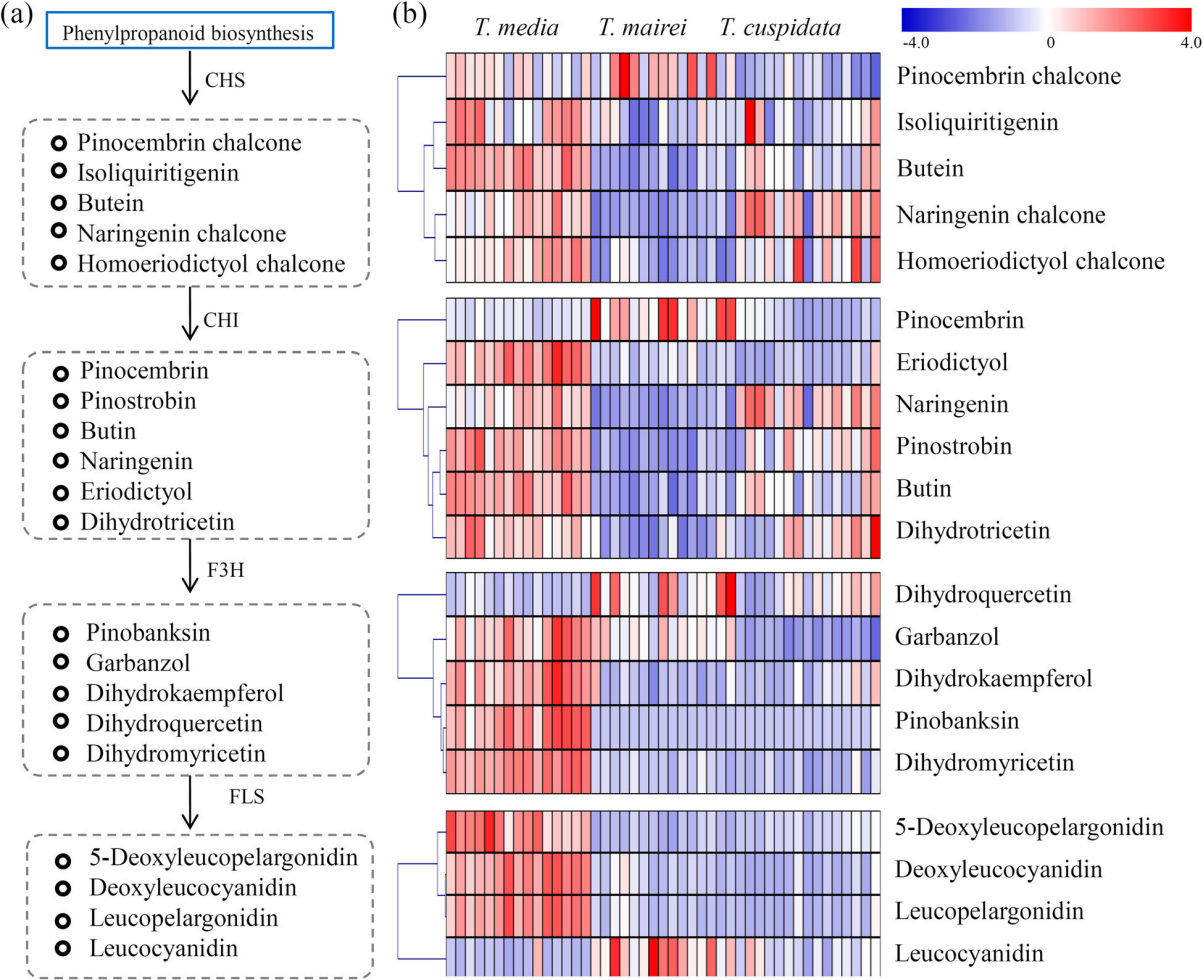
Analysis of the relative amounts of flavonoid in the Taxus metabolomes from the three different species. (**a**) Overview of the flavonoid biosynthesis pathway. (**b**) The accumulation levels of intermediate products synthesized by CHS, CHI, and F3H were showed by heatmaps. The heatmap scale ranges from -4 to +4 on a log_2_ scale

For flavonoid biosynthesis pathway, five intermediate products synthesized by chalcone synthase (CHS), six intermediate products synthesized by chalcone isomerase (CHI), five intermediate products synthesized by flavanone 3-hydroxylase (F3H), and four intermediate products synthesized by flavonol synthase (FLS) were identified (Fig. 5a). For the CHS-synthesized flavonoids, pinocembrin chalcone was highly accumulated in *T. mairei*, isoliquiritigenin, butein and homoeriodictyol chalcone were predominantly accumulated in *T. media*, and naringenin chalcone was greatly accumulated in both *T. media* and *T. cuspidata*. For the CHI-synthesized flavonoids, only pinocembrin was highly accumulated in *T. mairei*, eriodictyol and butin were largely accumulated in both *T. media*, and naringenin, pinostrobin and dihydrotricetin were predominantly accumulated in both *T. media* and *T. cuspidata*. Most of the F3H-synthesized flavonoids were predominantly accumulated in *T. media*, except for dihydroquercetin. For the FLS-synthesized flavonoids, 5-deoxyleucopelargonidin, deoxyleucocyanidin, and leucopelargonidin were highly accumulated in *T. media*, and leucocyanidin was greatly accumulated in *T. mairei* (Fig. 5b).

### Confirmation of the variations in paclitaxel and its derivatives using a targeted approach

**Fig. 6:**
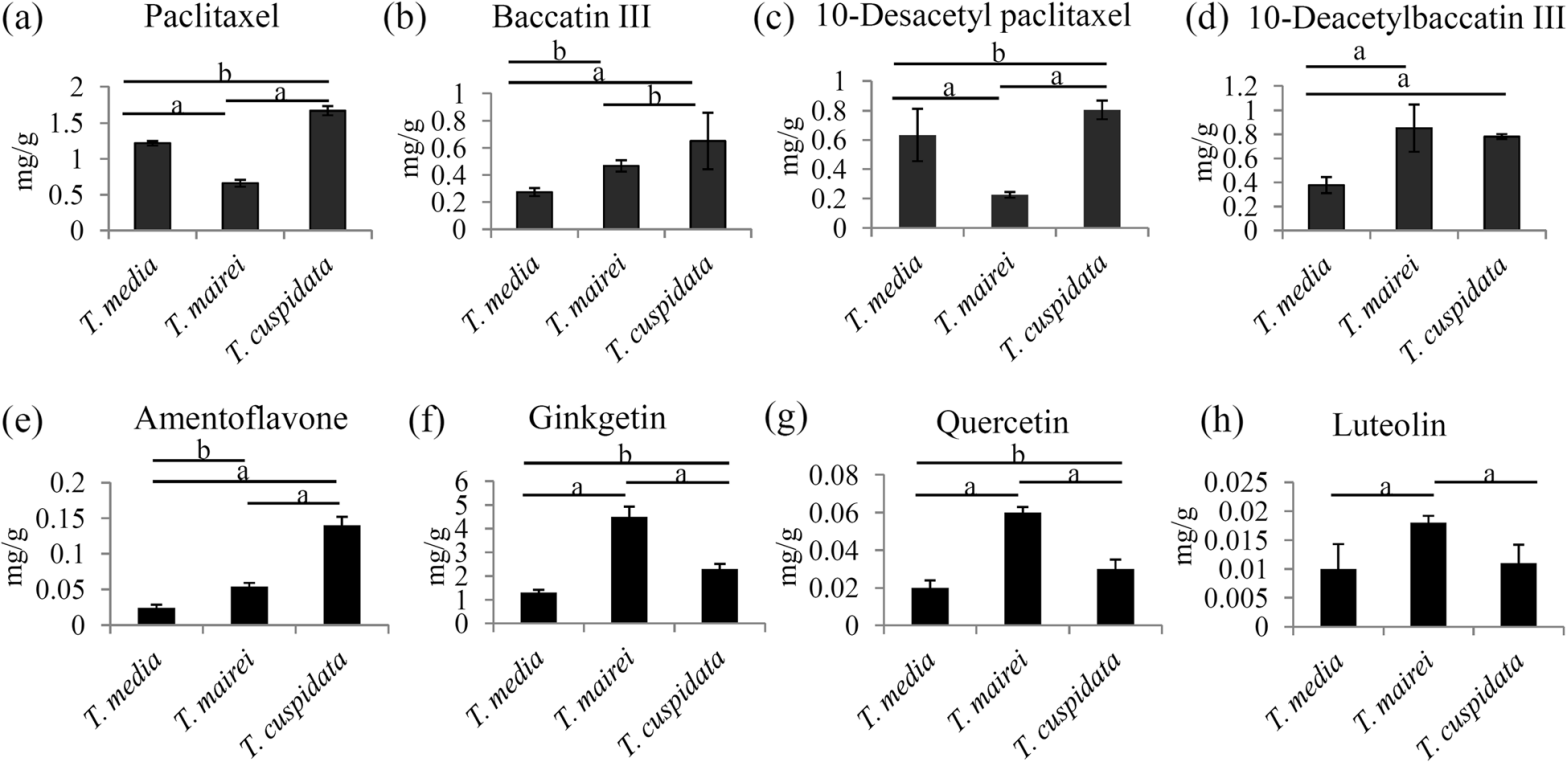
Variation of the contents of several selected taxoids and flavonoids among three different *Taxus* species**.** The contents of paclitaxel (**a**) and three intermediates, including baccatin III (**b**), 10-DAP (**c**), and 10-DAB III (**d**), were quantified by HPLC-MS/MS method. The contents of amentoflavone (**e**), ginkgetin (**f**), quercetin (**g**), and luteolin (**h**), were quantified by HPLC-MS/MS method. A *P* value < 0.05 was considered to be statistically significant and indicated by “b” and *P* < 0.01 was indicated by “a”

To determine more precisely the differences in taxoids among the three *Taxus* species, a targeted approach was used to measure the concentrations of paclitaxel, 10-DAB III, baccatin III, and 10-DAP (Additional file 7). The untargeted metabolomics analysis indicated that *T. cuspidata* and *T. mairei* contained the highest and the lowest levels of paclitaxel, respectively. The direct quantification with an authentic paclitaxel standard showed that *T. cuspidata*, *T. media*, and *T. mairei* contained 1.67 mg.g^− 1^, 1.22 mg.g^− 1^, and 0.66 mg.g^− 1^ of paclitaxel, respectively (Fig. 6a). The order of the paclitaxel contents was in good agreement with the untargeted metabolome results. For other taxoids, the highest levels of baccatin III and 10-DAP were accumulated in *T. cuspidata* (0.65 mg.g^− 1^ and 0.80 mg.g^− 1^, respectively), and the highest level of 10-DAB III was detected in *T. mairei* (0.85 mg.g^− 1^) (Fig. 6b-d). To assess variability in taxoid level among different species of the genus *Taxus*, another three *Taxus* species, including *T. chinensis*, *T. fuana* and *T. yunnanensis*, have been collected. A more exhaustive profile of taxoids in the genus has been showed in Additional file 8.

### Confirmation of the variations in flavonoids using a targeted approach

To determine more precisely the differences in flavonoids among the three *Taxus* species, a targeted approach was used to measure the concentrations of amentoflavone, ginkgetin, quercetin and luteolin (Additional file 9). Our data showed that amentoflavone highly accumulated in *T. cuspidata* (0.14 mg.g^− 1^) and lowly accumulated in *T. media* (0.024 mg.g^− 1^) (Fig. 6e). Interestingly, ginkgetin, quercetin and luteolin were greatly accumulated in *T. mairei* rather than the other two taxus trees (Fig. 6f-h).

### Systematic correlativity analysis identifies a number of metabolites associated with key metabolites of paclitaxel biosynthesis

**Fig. 7:**
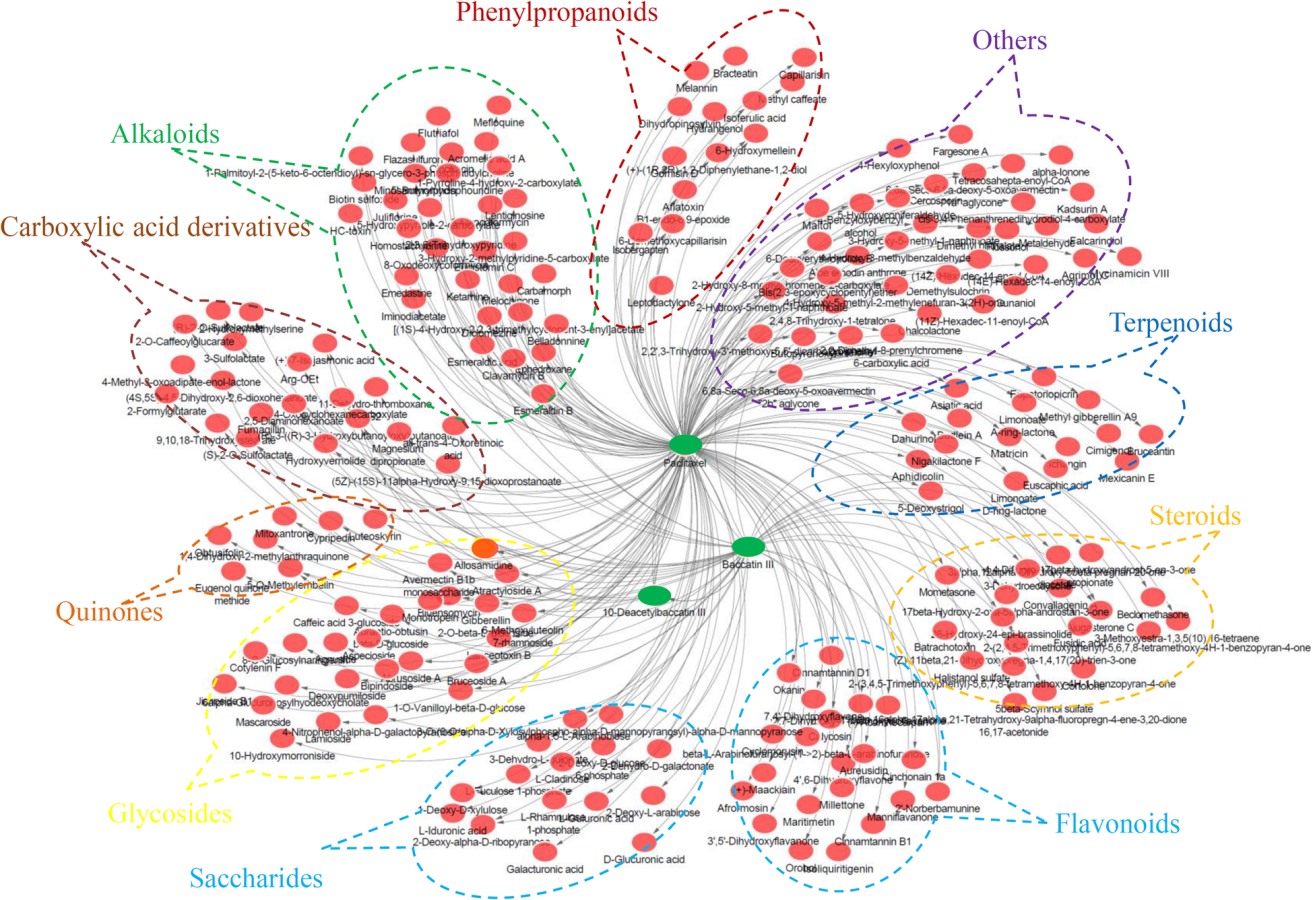
Analysis of metabolite-metabolite interaction networks. The taxoid-related networks were divided into three clusters surrounding paclitaxel, baccatin III and 10-DAB III, respectively. Nine major classes of metabolites grouped into various dotted circles with different color

An analysis of metabolite–metabolite interaction networks contributed to the understanding of functional relationships and the identification of new compounds associated with key metabolites of paclitaxel biosynthesis. In our study, an interaction network based on the differentially accumulated metabolites was constructed. Furthermore, the taxoid-related networks were divided into three clusters surrounding paclitaxel, baccatin III, and 10-DAB III (Additional file 10). The interaction networks suggested that nine classes of metabolites, phenylpropanoids, flavonoids, alkaloids, carboxylic acid derivatives, quinones, glycosides, saccharides, steroids and terpenoids, may also contribute to the variations in taxoid accumulation in different species (Fig. 7). However, the mechanisms underlying the interactions of these potential new metabolites need to be investigated.

## Discussion

Because *Taxus* plants are the major natural resource for paclitaxel, comprehensive phytochemical analyses of *Taxus* species have been performed [5, 25]. A large number of compounds have been identified in various *Taxus* species [7, 31]. In addition to taxane diterpenoids, many other compounds, including abietanes, lignans, polyprenols, phenolic compounds, and volatile components, were also identified in the twigs of *Taxus* plants [32–34]. However, the level of metabolite accumulation may vary significantly among species.

In plants, the accumulation of metabolites is a complex and important trait mainly affected by genetic and environmental factors [35, 36]. By identifying specific metabolites, our results suggested that variations, not only in paclitaxel and its derivatives, but also in their precursors, exist in different *Taxus* species (Fig. 3). The diterpenoid taxane core is derived by three units of IPP and one unit of dimethylallyl diphosphate, which are supplied by the MEP pathway [8]. Interestingly, most precursors for paclitaxel biosynthesis were highly accumulated in *T. mairei* compared with in *T. cuspidata*. For examples, three important intermediate products of the MEP pathway, including D-Glyceraldehyde 3P, 1-Deoxy-D-xylulose 5P and 2-C-Methyl-D-erythritol 4P, highly accumulated in *T. mairei*, ensuring the production of GGPP, which is a key precursor of the diterpenoid taxane core [8]. In our study, GGPP greatly accumulated in *T. mairei*, suggesting an abundant precursor supply in *T. mairei*. However, paclitaxel was primarily accumulated in *T. cuspidata* rather than in *T. mairei*. This suggested that the efficiency of paclitaxel synthesis using MEP pathway precursors in *T. cuspidata* may be extremely high.

The cyclization of the GGPP to taxa-4(5),11(12)-dien is an essential oxidation step on the taxane core [37]. Though taxa-4(5),11(12)-dien has not been detected, its hydroxylated products, taxa-4(20),11(12)-dien-5α-ol and taxa-4(20),11(12)-dien-5α,13α-diol, were identified and showed similar accumulation pattern to GGPP. In the taxol biosynthesis pathway, 10-DAB, a downstream product of taxa-4(20),11(12)-dien-5α-ol, is converted to baccatin III by 10-deacetylbaccatin III-10-O-acetyltransferase [12]. Moreover, CoA-dependent acyl transfers occur on the taxane core, yielding several acetylated intermediates, such as 10-deacetyl-2-debenzoylbaccatin III [38]. Interestingly, these acetylated products, taxa-4(20),11(12)-dien-5α-yl acetate, 10-deacetyl-2-debenzoylbaccatin III, 10-DAB and baccatin III, highly accumulated in *T. cuspidata* and *T. media*. Then, the attachment of β-phenylalanine to the C13-O-position of baccatin III to yield 3′-*N*-debenzoyl-2′-deoxytaxol and 3′-*N*-debenzoyltaxol, which are the direct upstream metabolites for taxol biosynthesis [39]. In our study, 3′-*N*-debenzoyl-2′-deoxytaxol and 3′-*N*-debenzoyltaxol highly accumulated in *T. cuspidata*. Most intermediate products approaching the end point of taxol biosynthesis pathway were primarily accumulated in *T. cuspidata*, suggesting there were higher-efficiency pathways to paclitaxel in *T. cuspidata* and *T. media* compared with in *T. mairei*.

Multiple anastomosing routes involved in the paclitaxel biosynthesis pathway produce numerous taxoid derivatives [5]. An approach to shutting down the major diversionary routes, such as the routes leading to 14β-hydroxy taxoids, and C9- and C13-acetate derivatives, could elevate paclitaxel yields [40, 41].

In addition to taxoids, flavonoids, phenylpropanoids, and phenolic compounds have been isolated in *Taxus* species [16, 31, 42, 43]. In our study, the metabolite–metabolite interaction network revealed 222 taxoid-associated metabolites, belonging to 10 major categories. In total, 21 flavonoids, including 3 baccatin III-related metabolites and 18 paclitaxel-related metabolites, were identified in the interaction network. Interestingly, the majority of the flavonoids were negatively correlated with baccatin III and paclitaxel (Additional file 10: Table S3), which was in accord with data from our metabolomes. A previous work showed that total flavonoids, ginkgetin, and quercetin were highly accumulated in *T. mairei* and that paclitaxel was highly accumulated in *T. media* [31]. Under ultrasound and salicylic acid treatments, paclitaxel biosynthesis improved and the flavonoid content significantly decreased [44]. These data suggested a negative correlation between paclitaxel biosynthesis and flavonoid metabolism.

A number of transcription factors (TFs) have been reported to be involved in the transcriptional regulation of taxol biosynthesis- and flavonoid biosynthesis-related genes [45, 46]. In plants, basic helix-loop-helix (bHLH) TFs were considered to be involved in flavonoids biosynthesis [47]. For example, DvIVS, a bHLH TF in dahlia, activates the flavonoid synthesis by regulating the expression of chalcone synthase 1, flavanone 3-hydroxylase, dihydroflavonol 4-reductase, anthocyanidin synthase [48]. In *Taxus* plants, three bHLH TFs, TcJAMYC1, TcJAMYC2 and TcJAMYC4, negatively regulate the expression of paclitaxel biosynthetic genes [49]. The opposite effects of bHLH TFs in regulations of flavonoid and taxol biosynthesis might give an explanation of negative correlation between paclitaxel biosynthesis and flavonoid accumulations. However, whether flavonoids were associated with paclitaxel biosynthesis needs to be addressed in the future.

In addition to the metabolites involved in the MEP pathway, 18 paclitaxel biosynthesis-associated terpenoids were identified. Inversion and homeostasis among terpenoids may play important roles in the precursor supply required for paclitaxel biosynthesis [8]. Because of the complexity of paclitaxel biosynthesis, more studies are needed to reveal the roles of the other identified metabolites.

## Conclusions

In our study, metabolic profiles reveal that the levels of metabolite accumulation may vary siginificantly among species. A large number of potential metabolites associated with paclitaxel biosynthesis were identified. Our results contribute to a deeper understanding of the interspecific differential accumulation of taxoids in three *Taxus* species.

## Methods

### Plant materials

Fresh twig samples were harvested from three-year-old cultivated *Taxus* trees, including *T. media*, *T. mairei*, and *T. cuspidata*, in March 2015 grown in a greenhouse of Hangzhou Normal University, Hangzhou, China. The growth conditions were set at 25 ± 1 °C with a light/dark cycle of 12/12 h and a 60–70% relative humidity.

### Metabolite extraction

For metabolite extraction, fresh twig samples from different *Taxus* species (25 mg each, *n* = 15) were transferred to 1.5-mL Eppendorf tubes, and 800 μL pre-cooled methanol/water (1:1, v/v) was added to the tube with two steel balls. All of the tubes were placed into a pre-cooled 48-well tube holder and ground using the 2010 Geno/Grinder (SPEX SamplePrep, Metuchen, NJ, USA) for 2 min at a rate of 1900 strokes/min. The homogenized samples were extracted in 0.5-mL of the pre-cooled chloroform/methanol/water (v:v:v, 1:3:1) extraction solvent by vortexing for 15 min at 4 °C in the dark and then ultrasonication for 5 min on ice. The samples were centrifuged at 13,000 g for 15 min at 4 °C, and 550 μL of the supernatants were collected. The extracts were vacuum-dried and resuspended in a 50% methanol solution. The prepared extracts were then loaded onto the auto-sampler of the 2777C ultra-performance liquid chromatography (UPLC) system (Waters, Herts, UK) at 4 °C.

### Untargeted metabolomic analysis

All of the samples were analyzed using the HPLC-MS/MS system. Firstly, the separation was achieved on a 100 × 2.1 mm, 1.7-μm particle size Waters ACQUITY UPLC BEH C18 column using an UPLC system (Waters, Herts, UK). The column oven was maintained at 50 °C, and the flow rate was set at 0.4 mL/min. The mobile phase consisted of solvent A (water with 0.1% formic acid) and solvent B (acetonitrile with 0.1% formic acid). Gradient elution conditions were set as follows: 100% phase A, 0–2 min; 0 to 100% phase B, 2–11 min; 100% phase B, 11–13 min; 0 to 100% phase A, 13–15 min. The injection volume for each sample was 10 μL.

A high resolution MS/MS Waters Xevo G2-XS Q-TOF (Waters, Herts, UK) was used to detect metabolites eluted from the column. The Q-TOF system was operated in both positive and negative ion modes. For the positive ion mode, the capillary and sampling cone voltages were set at 3 kV and 40 V, respectively. For the negative ion mode, the capillary and sampling cone voltages were set at 1 kV and 40 V, respectively. The MS data were acquired in centroid MSE mode. The mass range was from 50 to 1200 Da, and the scan time was 0.2 s. For the MS/MS detection, all of the precursors were fragmented using 20–40 eV, and the scan time was 0.2 s. During the acquisition, the LE signal was acquired every 3 s to calibrate the mass accuracy. To evaluate the stability of the UPLC-MS/MS system over the whole detection process, a quality control sample, which was prepared by mixing an equal volume of each experimental sample, was acquired after every 10 samples.

### Bioinformatics of the untargeted metabolomic dataset

Raw data of UPLC-MS/MS were processed using the following procedures. For each sample, a matrix of molecular features, such as retention time and mass-to-charge ratio (*m/z*), was generated using the XCMS software with default parameters [50]. The data were normalized to the total ion current, and the relative quantity of each feature was calculated using the mean area of the chromatographic peaks from three replicate injections. The quantities of metabolites were generated using an algorithm that clustered masses into spectra based on co-variation and co-elution in the dataset. The online Kyoto Encyclopedia of Genes and Genomes (KEGG) and HMDB database was used to annotate the metabolites by matching the exact molecular mass data (*m/z*). If a mass difference between observed and the database value was less than 10 ppm, the metabolite would be annotated and the molecular formula of metabolites would further be identified and validated by the isotopic distribution measurements. We also used a in-house fragment spectrum library of metabolites to validate the metabolite identification. The intensity of peak data was further processed by an in-house software MetaX. For quality control, the identifications of precursor ions of the expected positive ion adduct with less than a 5 ppm error were defined using high-resolution MS. The raw data were uploaded as Additional file 11 and Additional file 12.

### K-means cluster

ClusGap R function-cluster package (v.2.0.5) was used to determine the optimal number of clusters. Subsequently, K-means clustering with default algorithm was used to get clusters using the scaled normalized relative metabolite data on a log2 scale for each accumulated metabolite. The results of clustering were displayed using MeV program.

### Analysis of targeted metabolites

Fresh twigs of each sample were collected from three *Taxus* species, dried at 40 °C for 3 d, and powdered. A modified version of a previously published method was used to prepare crude extracts [51]. In brief, 2.0 g powder of each sample was mixed with 30 mL of 100% methanol, and the mixture was subjected to ultrasonication for 60 min. After centrifugation at 5000 g for 5 min, the supernatant was filtered through 0.22-μm membrane filters and transferred to a new tube.

The quantifications of four targeted taxoids, paclitaxel, BAC, 10-DAB III, and 10-Desacetyl paclitaxel (10-DAP), were performed using HPLC-MS/MS analyses. Paclitaxel (≥ 99%; CAS No. 33069–62-4), baccatin III (≥ 99%; CAS No. 27548–93-2), and 10-DAB III (≥ 98%; CAS No. 32981–86-5) were purchased from Aladdin Biochemical Technology (Shanghai, China). 10-DAP (98%; CAS No. 78432–77-6) was obtained from the Jiangsu Yew Pharmaceutical Co., Ltd. (Jiangsu, China).

Taxoids were detected using a Thermo Dionex UltiMate 3000 series HPLC system equipped with a Finnigan TSQ Quantum Discovery triple quadrupole MS (Thermo Fisher Scientific, Waltham, MA, USA). The separation of the above four compounds was carried out on a Phenomenex Kinetex C18 column (100 × 4.6 mm, 2.6-μm particle size; Phenomenex, Torrance, CA, USA). The mobile phase consisted of 35% of solvent A (2 mM ammonium formate and 0.1% formic acid aqueous solution) and 65% of solvent B (100% methanol). The flow rate was 0.2 mL/min, the temperature of column oven was 30 °C, and the injection volume was 5 μL. Other detailed parameters of the HPLC-MS/MS analysis were as follows: the capillary temperature was 270 °C; the ion spray voltage was 3000 V; the auxiliary gas and sheath gas was N_2_; and the collision gas was high purity argon. Additionally, the positive electrospray ionization mode was employed and multiple-reaction monitoring was applied for the determination. The transition of *m/z* 567.2 → 445.3 was used for 10-DAB III quantification, and the transitions of *m/z* 567.2 → 385.2 and 567.2 → 427.3 were utilized for confirmation. The transitions of *m/z* 829.4 → 286.1 and 829.4 → 122.0 were measured for baccatin III quantification and confirmation, respectively. The transition of *m/z* 876.4 → 308.1 was chosen for paclitaxel quantification, and the transitions of *m/z* 876.4 → 531.2 and 876.4 → 591.4 were utilized for confirmation. The transition of m/z 834.4 → 308.2 was used for 10-Desacetyl paclitaxel quantification. Data was acquired and processed using the Xcalibur 2.2 software (Thermo Scientific, Waltham, MA, USA).

The separation and determination of eight flavonoids, including quercetin, luteolin, kaempferol, amentoflavone and ginkgetin, were performed according to the same UPLC-MS/MS method described in our paper [51].

### Systematic correlativity analysis and statistical analysis

For the untargeted metabolome analyses, Pearson’s and Spearman’s correlations, a one-way analysis of variance (ANOVA), and hierarchical clustering were conducted. *P* values of the ANOVA were adjusted for the false discovery rate. A principal component analysis (PCA) of the metabolites was performed on the data that was mean-centered with the Pareto-scaling method using SIMCA v14.0 (Umetrics, Umea, Sweden).

The quantification results of targeted metabolites are presented as the means of at least three replicates ± standard error. Statistical analyses were performed using SPSS software version 19.0 (SPSS Inc., Chicago, IL, USA), and an ANOVA was applied to compare taxoid content differences. A *P* value < 0.05 was considered to be statistically significant.

## Supplementary information

**Additional file 1: Table S1.** Detail information of 2246 identified metabolites.

**Additional file 2: Figure S1.** The total ion chromatograms of all the samples.

**Additional file 3: Table S2.** The KEGG annotations of 747 identified metabolites.

**Additional file 4: Figure S2.** Identification of DAMs in the *T. mairei* vs *T. cuspidata* comparisons. The box indicated the ‘diterpenoid biosynthesis’ pathway.

**Additional file 5: Figure S3.** Identification of DAMs in the *T. media* and *T. mairei* comparison. The box indicated the ‘diterpenoid biosynthesis’ pathway.

**Additional file 6: Figure S4.** Identification of DAMs in the *T. media* and *T. cuspidata* comparison. The box indicated the ‘diterpenoid biosynthesis’ pathway.

**Additional file 7: Figure S5.** Chromatograms of individual taxoids.

**Additional file 8: Figure S6.** A more exhaustive profile of taxoids in the *Taxus* genus.

**Additional file 9: Figure S7.** Chromatograms of individual flavonoids.

**Additional file 10: Table S3.** The important information of taxoid-related networks.

**Additional file 11:** Dataset 1 Metabolomic input raw data 1.

**Additional file 12:** Dataset 2 Metabolomic input raw data 2.

## Data Availability

All the datasets generated and analysed during the current study were uploaded as with the manuscript as additional files.

## Abbreviations

10-DAB III: 10-deacetylbaccatin III
10-DAB: 10-deacetylbaccatin-III
ANOVA: Analysis of variance
DAM: Differential accumulated metabolite
DMAPP: Dimethylallyl diphosphate
GGPP: Geranylgeranyl diphosphate
HPLC-MS/MS: high-performance liquid chromatography-tandem mass spectrometry
IPP: Isopentenyl diphosphate
JA: Jasmonic acid
MEP: 2-C-methyl-D-erythritol phosphate
PCA: Principal component analysis
TS: Taxadiene synthase
