# Supplementary figures and images for: Comparative metabolomic analysis reveals the variations in taxoids and flavonoids among three Taxus species
Source: BMC Plant Biol. 2019 Nov 29;19:529. doi: 10.1186/s12870-019-2146-7 (PMC6884900; doi:10.1186/s12870-019-2146-7)

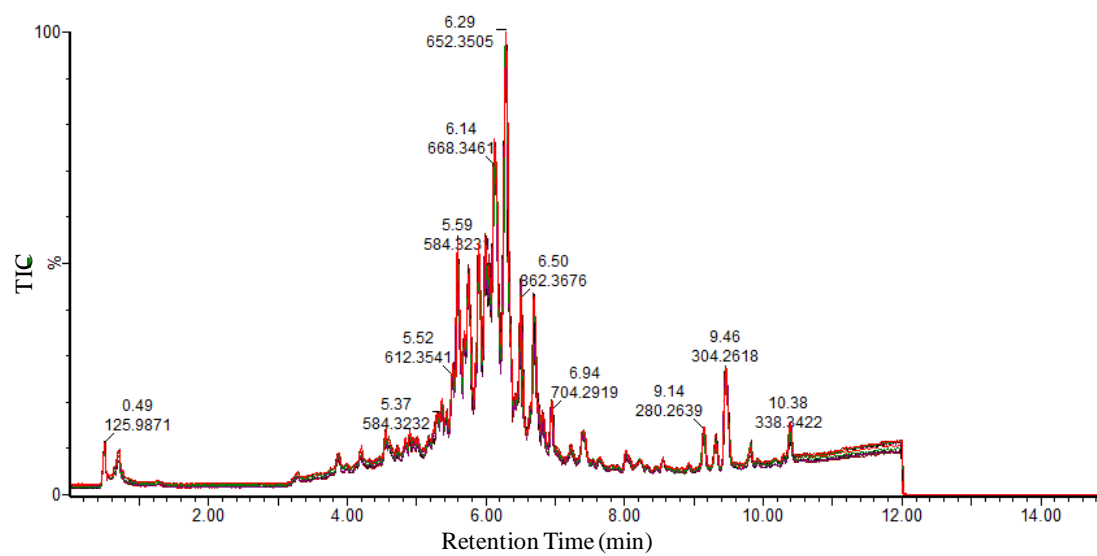

Figure S1 The total ion chromatograms of all the samples.

Supplement: Supplementary file 2 — Additional file 2: Figure S1. The total ion chromatograms of all the samples. [file 12870_2019_2146_MOESM2_ESM.pdf]

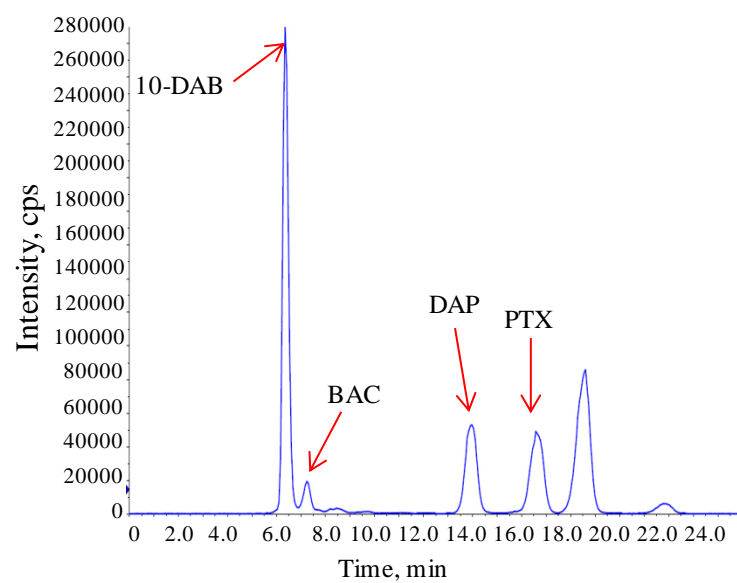

Figure S5 Chromatograms of individual taxoids.

Supplement: Supplementary file 7 — Additional file 7: Figure S5. Chromatograms of individual taxoids. [file 12870_2019_2146_MOESM7_ESM.pdf]

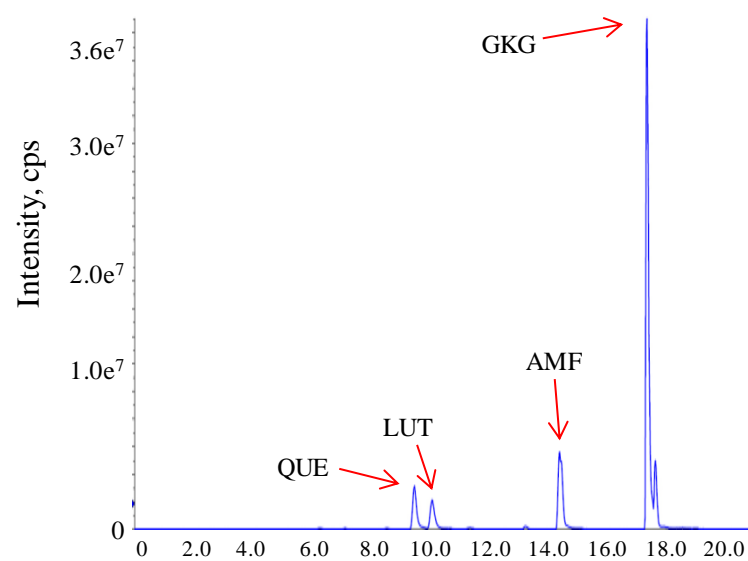

Figure S7 Chromatograms of individual flavonoids.

Supplement: Supplementary file 9 — Additional file 9: Figure S7. Chromatograms of individual flavonoids. [file 12870_2019_2146_MOESM9_ESM.pdf]
