# Supplementary material for: Comparative metabolomic analysis reveals the variations in taxoids and flavonoids among three Taxus species
Source: BMC Plant Biol. 2019 Nov 29;19:529. doi: 10.1186/s12870-019-2146-7 (PMC6884900; doi:10.1186/s12870-019-2146-7)
Supplement: Supplementary file 4 — Additional file 4: Figure S2. Identification of DAMs in the T. mairei vs T. cuspidata comparisons. The box indicated the ‘diterpenoid biosynthesis’ pathway. [file 12870_2019_2146_MOESM4_ESM.pdf]

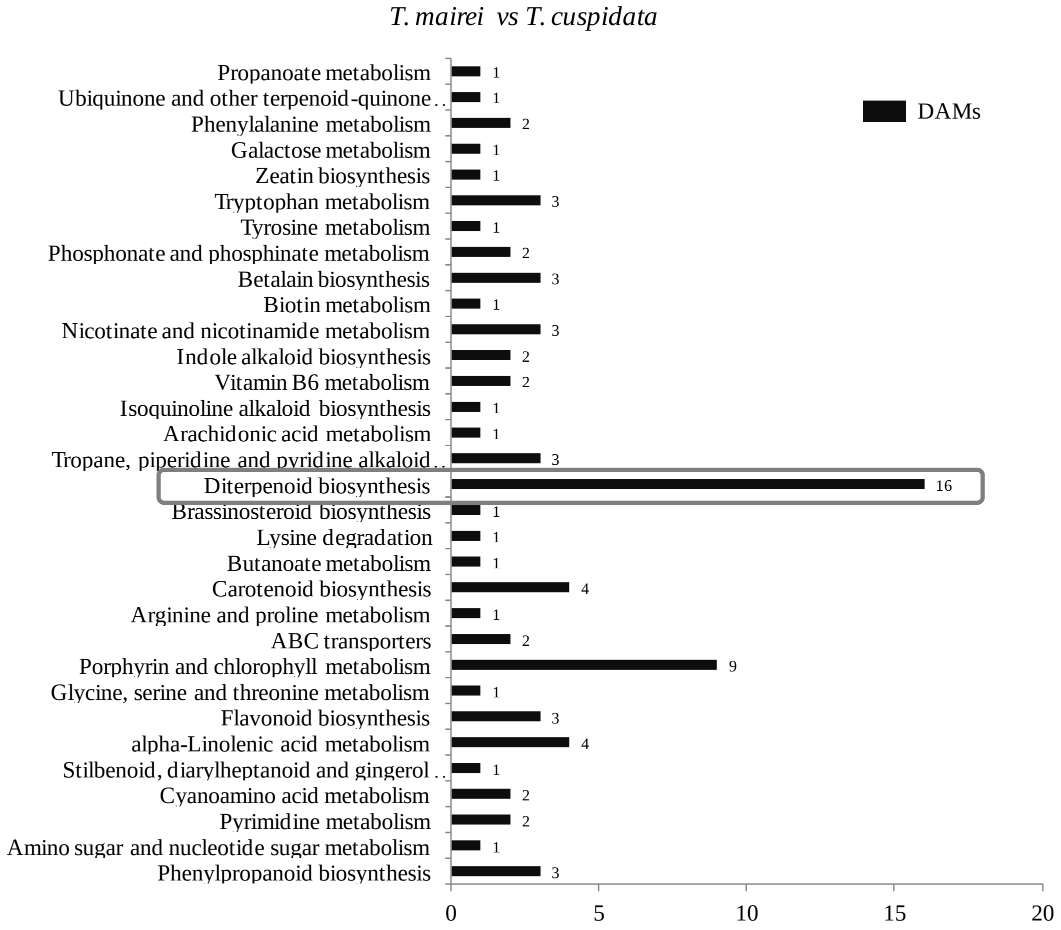

Figure S2 Identification of DAMs in the *T. mairei* vs *T. cuspidata* comparisons. The box indicated the ‘diterpenoid biosynthesis’ pathway.
