# Supplementary material for: Comparative metabolomic analysis reveals the variations in taxoids and flavonoids among three Taxus species
Source: BMC Plant Biol. 2019 Nov 29;19:529. doi: 10.1186/s12870-019-2146-7 (PMC6884900; doi:10.1186/s12870-019-2146-7)
Supplement: Supplementary file 6 — Additional file 6: Figure S4. Identification of DAMs in the T. media and T. cuspidata comparison. The box indicated the ‘diterpenoid biosynthesis’ pathway. [file 12870_2019_2146_MOESM6_ESM.pdf]

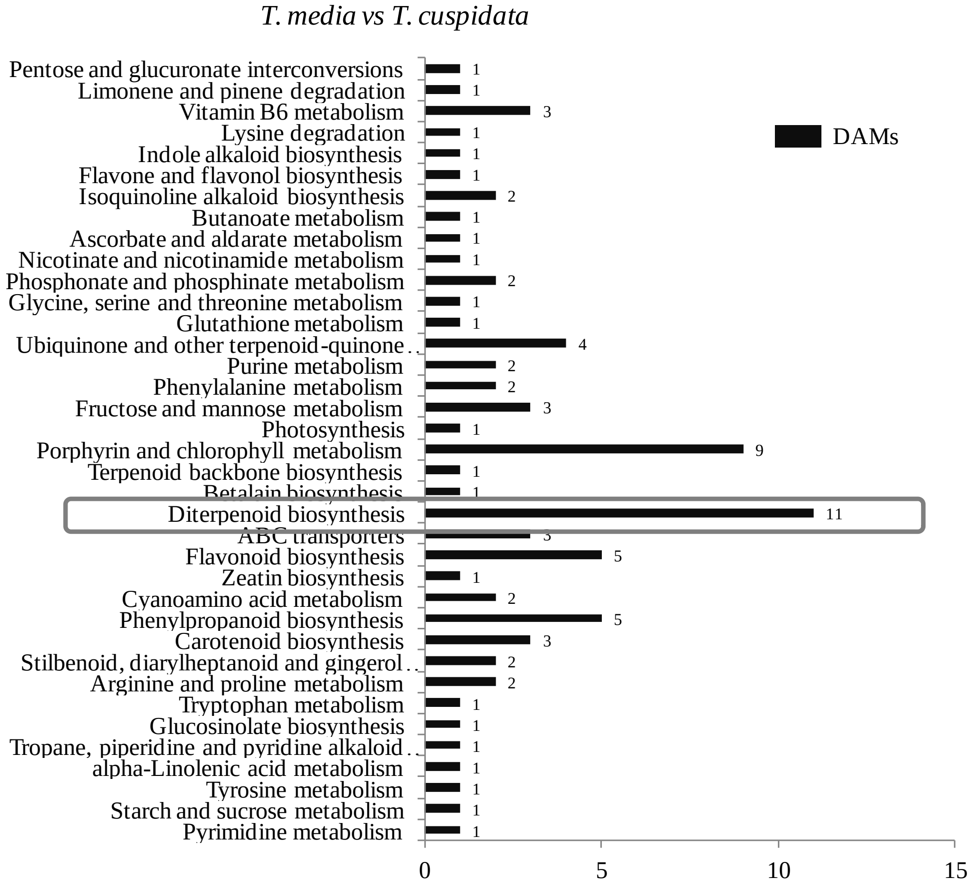

Figure S4 Identification of DAMs in the *T. media and T. cuspidata* comparison. The box indicated the ‘diterpenoid biosynthesis’ pathway.
