# Supplementary material for: Comparative metabolomic analysis reveals the variations in taxoids and flavonoids among three Taxus species
Source: BMC Plant Biol. 2019 Nov 29;19:529. doi: 10.1186/s12870-019-2146-7 (PMC6884900; doi:10.1186/s12870-019-2146-7)
Supplement: Supplementary file 8 — Additional file 8: Figure S6. A more exhaustive profile of taxoids in the Taxus genus. [file 12870_2019_2146_MOESM8_ESM.pdf]

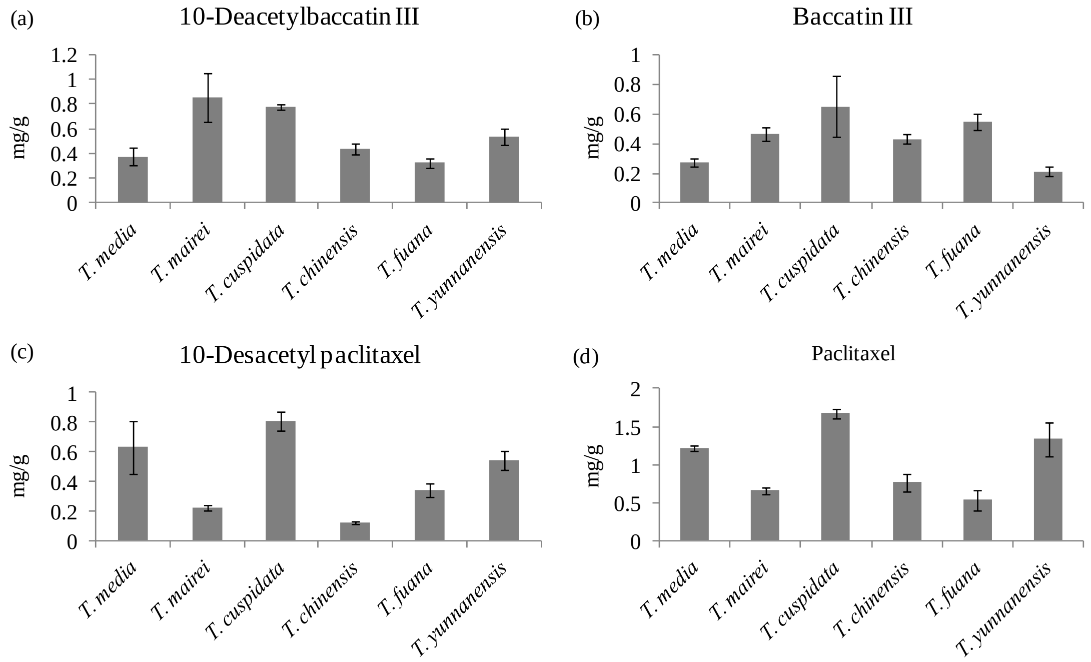

Figure S6 **A more exhaustive profile of taxoids in the *Taxus* genus.** The contents of three intermediates, including 10-DAB III (a), baccatin III (b), , and 10-DAP (c), and paclitaxel (d) were quantified by HPLC-MS/MS method.
